# Perception of graduates about the education of Nursing Technicians

**DOI:** 10.1590/0034-7167-2022-0325

**Published:** 2023-10-06

**Authors:** Gabriel Luiz Nascimento Fioramonte, Adriana Avanzi Marques Pinto, Maria José Sanches Marin

**Affiliations:** I Faculdade Solidária de Presidente Prudente. Presidente Prudente, São Paulo, Brazil; II Fundação Educacional do Município de Assis. Assis, São Paulo, Brazil; III Faculdade de Medicina de Marília. Marília, São Paulo, Brazil

**Keywords:** Education, Education, Nursing, Associate, Learning, Health Human Resource Training, Professional Training, Educación, Graduación En Auxiliar De Enfermería, Aprendizaje, Capacitación De Recursos Humanos En Salud, Capacitación Professional, Educação, Educação Técnica Em Enfermagem, Aprendizagem, Capacitação De Recursos Humanos Em Saúde, Formação Profissional

## Abstract

**Objectives:**

to interpret the perception of graduates from the nursing technician program on the learning process developed during their education.

**Methods:**

a qualitative study was conducted from March to September 2021, based on interviews with 20 graduates from a nursing technician program at a school in the Midwest of São Paulo, using thematic analysis and the NVivo tool.

**Results:**

the following themes were identified: traditional teaching methods, active learning strategies, valuing proactivity and experiences in professional practice.

**Final Considerations:**

according to the graduates, the teaching process is essentially based on traditional methods, although there are initiatives to implement active strategies and recognition of the importance of advancing student proactivity and practical experience.

## RESUMO

**Objetivos:**

interpretar a percepção de egressos do curso técnico de enfermagem sobre o processo de aprendizagem desenvolvido durante sua formação.

**Métodos:**

estudo qualitativo realizado no período de março a setembro de 2021, a partir de entrevistas com 20 egressos do curso técnico de enfermagem de uma escola do Centro-Oeste paulista, por meio da análise temática, utilizando a ferramenta *NVivo*.

**Resultados:**

foram identificados os temas: método tradicional de ensino, estratégias ativas de aprendizagem, atribuição de valor à proatividade e às vivências na prática profissional.

**Considerações Finais:**

para os egressos, o processo de ensino encontra-se essencialmente pautado no método tradicional, embora existam iniciativas de implantação de estratégias ativas e reconhecimento da importância de avançar na proatividade do estudante e aproximação com a prática.

## RESUMEN

**Objetivos:**

interpretar la percepción de egresados del curso técnico de enfermería sobre el proceso de aprendizaje desarrollado durante su formación.

**Métodos:**

estudio cualitativo realizado en el periodo de marzo a septiembre de 2021, a partir de entrevistas con 20 egresados del curso técnico de enfermería de una escuela del Centro-Oeste paulista, mediante el análisis temático, utilizando la herramienta NVivo.

**Resultados:**

se identificaron los temas: método tradicional de enseñanza, estrategias activas de aprendizaje, atribución de valor a la proactividad y a las experiencias en la práctica profesional.

**Consideraciones Finales:**

para los egresados, el proceso de enseñanza se encuentra esencialmente basado en el método tradicional, aunque existen iniciativas de implantación de estrategias activas y reconocimiento de la importancia de avanzar en la proactividad del estudiante y acercamiento con la práctica.

## INTRODUCTION

For the Brazilian reality, reflecting on the education of nursing technicians is of great relevance, as they represent a large contingent of professionals working in the healthcare field. They are present in all care settings and, most of the time, are responsible for direct contact with users, which is the space where care is effectively delivered and quality is expressed positively or negatively.

In this context, when analyzed from the perspective of nurses working in hospital services, this education shows that these professionals enter the job market without the knowledge, attitudes, and skills inherent to their professional role. Therefore, it is necessary to undertake differentiated teaching and learning processes, suggesting that pedagogical planning should involve professionals from healthcare services, with more rigorous evaluation processes, better teacher training, and an increase in the internship workload^(1)^.

Investments in the education of these professionals have been highlighted since the 1980s, mobilized by educational and healthcare policies, as well as Law 7.498/86, which abolished the nursing assistant position, as it represented a large contingent of professionals working in the hospital field without proper training^(2)^.

The Large-Scale Training Project for Medium-Level Personnel is an innovative initiative that aimed to prepare professionals for the Unified Health System (SUS), according to the definitions established by the VIII National Health Conference, which directed the need for the reformulation of health services and expansion of access. This project was developed in connection with the health production process and the use of problem-based learning^(3)^.

In 1999, based on the Large-Scale Training Project, the federal government created the Professionalization Program for Nursing Workers (PROFAE), with the purpose of qualifying professionals in healthcare services. In 2000, the Technical Schools of Nursing Network was created, with training centers in all Brazilian states^(4)^.

Despite these movements, imbued with transformative principles of social reality, they were not sufficient to meet the growing demand, especially for those who see the profession as a way to have a professional qualification and entry into the job market. Therefore, it is observed that private initiatives are given space, which, even following curricular guidelines, are isolated and offer few possibilities and little openness for reflection and transformation of teaching processes^(5)^.

The current National Curriculum Guidelines for vocational technical education at the high school level propose, among other aspects, the inseparability between education and social practice, the valorization of subjects, student-centered learning, overcoming knowledge fragmentation, curriculum contextualization and flexibility, as well as interdisciplinary approaches, aiming at overcoming the existing fragmentation in the teaching process^(6)^.

Therefore, innovations are necessary to develop professionals with autonomy, proactivity, and critical thinking. Active learning methodologies are highlighted in this sense, which are based on significant discovery learning and principles of critical/reflexive conception. Essentially, these methodologies aim to stimulate students’ creativity and autonomy in solving problems identified in their professional practice experiences^(7)^. To achieve this, students must be prepared to fully use their psychological, cognitive, and socioemotional functions, aiming to face new situations with dynamism, flexibility, and creativity, understanding their social, economic, technological, and scientific foundations. This will enable students to play an active role in learning and attribute meaning to it^(8)^.

However, despite the progress regarding the use of active learning methodologies, the influence of the traditional teaching method focused on the teacher and content transfer remains frequent, where students remain passive listeners, that is, they only receive and memorize content in a reproductive behavior^(9)^.

Thus, it can be inferred that the education of nursing technicians presents both advances and challenges. Despite its great relevance, the literature has a limited number of scientific publications that address this education, mainly from the perspective of the actors in the process^(10)^. It is therefore necessary to conduct research on this topic, aiming to highlight the paths being taken to make this education adequate to social needs. In this study, we seek to bring visibility and enable reflections on the challenges of this education^(10)^.

## OBJECTIVES

To interpret the perception of graduates from a nursing technician course about the learning process developed during their education.

## METHODS

### Ethical aspects

The project was submitted to the Ethics and Research Committee on Human Beings of the proposing institution, in order to ensure its adequacy to the ethical norms for research involving human beings. To preserve the anonymity of the interviewees, they were identified by the letter “I” followed by the numerical order of realization, from I1 to I20. All participants returned to the researcher, via email, the Consent Terms duly signed in accordance with Resolution 510/16 and Circular Letter No. 1/2021-CONEP/SECNS/MSo1/2021.

### Study type

This is a qualitative field study, based on thematic analysis proposed by Braun and Clark, carried out from semi-structured interviews with graduates from a nursing technician course^(11)^. To guide the methodology of this study, the Consolidated Criteria for Reporting Qualitative Research (COREQ)^(12)^ instrument was used.

### Study Setting

The research was conducted with graduates from a for-profit school of technical vocational courses in a municipality located in the western region of the State of São Paulo, which offers preschool, elementary, and high school education in the daytime and afternoon periods, and vocational education in the evening period. The school offers 70 places for the technical nursing course per semester, which, when filled, form two classrooms with approximately 35 students each.

### Data Source

The study participants are graduates from the years 2017 to 2019, with 98 from 2017, 91 from 2018, and 102 from 2019, totaling 292 graduates. Convenience sampling was used to select participants. Contact with graduates was made through social networks, mainly Facebook. As each participant was interviewed, they were asked to indicate one or two friends. At least three contacts were made for each indication. In the absence of a response, a new graduate was sought.

Inclusion criteria were graduates who had not worked as nursing assistants before entering the technical course, and exclusion criteria were those who were not working in the nursing field. The sample size was determined by saturation, that is, at the point when it was considered that new data would not add to the study’s objective, since it already had population heterogeneity and sufficient data volume and richness of information, as well as repetition of ideas observed in the latest interviews^(13)^. Thus, 20 graduates were interviewed, with only one interview conducted per participant. It is worth noting that 15 contacted graduates refused to participate, many of them citing a lack of availability.

### Data Collection and Organization

Considering the social isolation period due to the Covid-19 pandemic, interviews were conducted remotely using applications such as WhatsApp, Zoom Platform, Google Meet, and Skype, which were recorded for subsequent transcription and analysis. Through WhatsApp, it was possible to make video and voice calls, with only the participant and researcher present during the interview.

The interviews occurred from March to September 2021. To conduct them, a script was developed containing identification data such as gender, age, and marital status; professional data such as time of graduation and workplace, in addition to the following open-ended questions: “During your education, what forms/resources did teachers use to teach classes? Do you remember any different strategy from the lecture that they used? What do you think about how the teaching process was carried out? Talk about if you have any improvement proposal for the way teaching was developed in your course”.

Field notes were made immediately after the interviews, where the researcher recorded impressions of the participants’ emotional expressions. After the first contact with the graduates, which was done through social networks or colleague referrals, an email was requested for sending the invitation containing the explanation about the study and the importance of participation in the research, and attached was the Informed Consent Form, which was signed and returned to the researcher. The email also requested the definition of date, time, and the most appropriate means for conducting the interview.

The interviews had an average duration of 30 minutes and were conducted by the principal investigator, who is a nurse, a master’s student, and received the necessary training to develop the technique. Considering the difficulties of contacting the participants due to the lack of availability of time and the pandemic period, the transcriptions were not validated by them. The interviews were coded with the letter “E”, following their order of execution (E1...E20).

For the analysis of the obtained data, the thematic analysis technique was chosen, which is a qualitative analytical method that seeks to identify, analyze, and report patterns (themes) within the data and interpret various aspects of the research theme. In this form of analysis, the themes are extracted from the data itself and represent something important that is abstracted from the data in relation to the study’s purpose, which may or may not appear with great prevalence in the set of information.

Considering that the development of themes occurs from successive approximations with the data, six steps are proposed for this form of analysis, with the guidance that rich and complex insights must be generated. The steps are as follows: familiarity with the data, which involves immersion in the data through readings, aiming for a deep and broad approach to the content; initial code production from the data, which refers to the most basic segment or element of the data; searching for themes, which, in turn, is developed from the list of codes, involving the screening of different codes into potential themes; revisiting the themes, which involves their refinement, taking into account the criteria of internal homogeneity and external heterogeneity; definition and naming of the themes constitute the moment when the essence of each theme’s subject is identified, and as part of the refinement, it is identified whether a theme has sub-themes; final analysis and report writing^(11)^.

To proceed with the analysis, NVivo Plus software, version 11, was used as a tool. Initially, the 20 interviews were inserted, and then the initial codes referring to each nucleus of meaning found were created, with subsequent reviews and groupings to arrive at the final themes. Conceptual maps were developed within the software to assist in data systematization. Although there are different types of qualitative analysis software on the market, NVivo is pointed out to aggregate important features contained in other packages^(14)^. The data coding was analyzed and approved by the three researchers.

### Analysis of data

The sample was characterized by 20 graduates, the majority of whom were female, with 13 participants (65%) and seven males (35%). The age group of the interviewees with the highest predominance was 24 to 29 years old, with eight (40%), followed by 18 to 23 years old with seven (35%). The most notable length of work experience was between 1 to 3 years with 13 (65%), and the second most common length of work experience was over 4 years with five (25%). It is also possible to verify from the sociodemographic data that the time taken to enter the job market with the highest results was less than or equal to 1 month, with nine graduates (45%), while the second was between 1 to 3 months with seven (35%).

**Figure 1:**
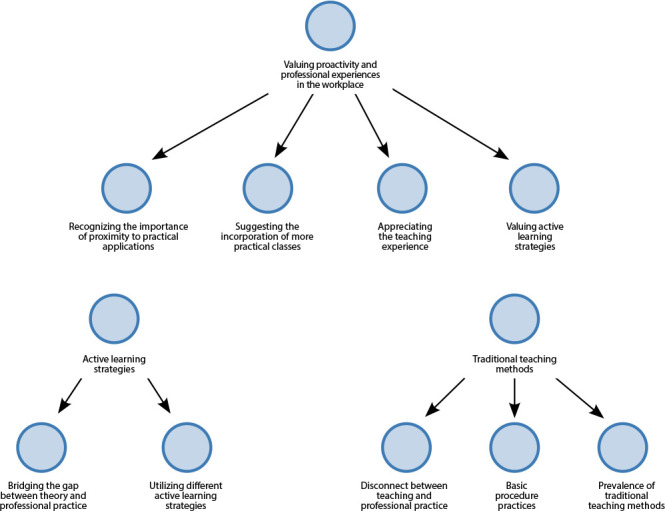
Distribution of themes and respective subthemes, according to the model generated by NVivo, Marília, São Paulo, Brazil, 2022

In the analysis of the interviews, as observed in Figure 1, three themes were identified that relate to the traditional teaching method, active learning strategies, and the attribution of value to proactivity and experiences in professional practice.

**Figure 2:**
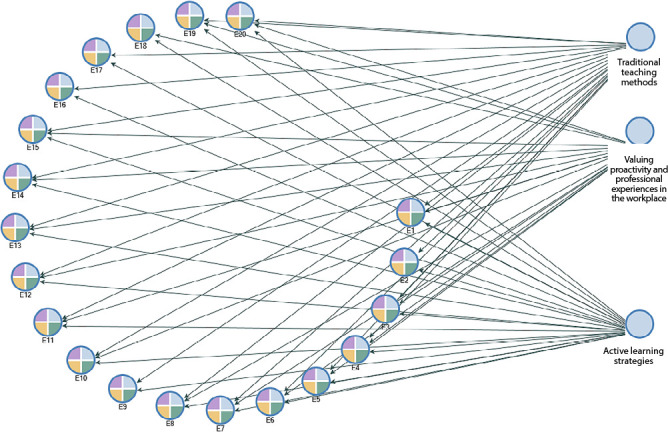
Relationship between themes and interviewees, according to the model generated by NVivo, Marília, São Paulo, Brazil, 2022

In Figure 2, the relationship between the elaborated themes and study participants is presented, revealing the totality of themes that were addressed by the majority of participants.

## RESULTS

### Dominance of traditional method

Regarding the theme “traditional teaching method,” it is evident in the statements of the graduates that this method predominated during their education. This is expressed through the use of handouts prepared by the teacher, the use of chalkboards, and repeated explanations, which leads to memorization of the content.

[...] *The teachers used to provide study materials in the form of a booklet, on the blackboard, or by handing out some kind of brochure, and each teacher who taught their subject would put together their own study materials.* (E10)

*The teachers used to explain the content thoroughly, and if necessary, we could review the materials in the study materials; there were some teachers who would repeat the same thing so many times that we ended up memorizing it.* (E16)

[...] *but mostly it was the teacher standing at the front (of the classroom) and us sitting and listening or participating* [...]. (E11)

The interviewees also mention the development of basic procedures, suggesting that they are performed in an isolated and decontextualized manner.

*Actually, we had some classes on basic techniques where we had hands-on practice, like putting on sterile gloves, taking blood pressure, checking vital signs, and we did it inside the classroom.* (E3)

[...] *the teacher would demonstrate how to perform basic techniques and explain, while we practiced on other students.* (E8)

Regarding the context of traditional teaching, the interviewees note that there was a gap between theory and practice during their education. Additionally, they believe that true learning occurs in everyday life, in line with existing practices in this setting.

[...] *We see a lot of theory in books, everything looks nice, but when we get to practice it’s all different* [...]. (E10)

[...] *theory is very different from practice, we learn more on a day-to-day basis, but when you do the course you get an idea, I learn every day inside the hospital because it’s very different, we learn the way things are done in the hospital.* (E7)

### Active learning strategies

Although the learning process was based on traditional teaching methods, the interviewees reported initiatives indicating the use of active learning strategies, as well as a closer integration of theory and practice. In relation to active learning strategies, the graduate mentioned that the introduction of dynamics using dolls for training facilitates learning. The case study was also used as a way to enliven the debate on a particular topic.

[...] *they used dolls, we practiced on ourselves, for me, these classes were very important, it was much easier to learn than when we only had the textbook.* (E3)

[...] *they brought some case studies for us to read and discuss about that subject, there were subjects where we did the technique practice* [...]. (E15)

For the interviewees, the integration of theory with professional practice occurred through demonstrations and doing things together.

[...] *besides the explanations, they showed us how to do it, so we did it together, there was an attempt to combine practice with theory.* (E6)

[...] *oh, they used learning methods that tried to show it as close as possible to reality, it made our learning easier.* (E7)

### Value assigned to proactivity and practical experiences

Even though they were trained predominantly by traditional methods, the interviewees indicate the need for more practical classes. They value teaching experience, active learning strategies, as well as integration with professional practice. Thus, they suggest the inclusion of more practical classes, even if in a laboratory, as it would be a way to expand learning.

[...] *if there were more practical laboratory classes, learning would be easier* [...]. (E13)

[...] *we could have more moments to perform venous puncture* [practical class]*. An improvement proposal would be to increase practical classes.* (E18)

The graduates indicate as positive the fact that the coordination seeks teachers with experience in the field, and consider that they are always seeking more effective ways to develop the teaching process.

*The coordination team looks for professionals who work in the area, so it was very complete* [...]. (E4)

*The teachers are always looking for a better way to teach.* (E1)

From the interviewees’ speech, it is evident that they value and suggest the inclusion of strategies capable of promoting information exchange, autonomy, and protagonism in teaching.

[...] *even though I didn’t like it, these presentation assignments also help because* [...] *if I close my eyes, I can remember my presentations, and there are many classes that I don’t remember.* (E15)

*I think we should talk about a topic, do a practice, have a discussion about it. Give a few minutes to discuss with colleagues, or try to explain what you learned to a colleague.* (E3)

The graduates also recognize the importance of closer integration of theory and practice, even before entering the internship field.

[...] *trying to associate theory with practice would be really nice, because it’s not just about going to the hospital and doing an internship.* (E20)

## DISCUSSION

The present study aimed to analyze, from the perspective of graduates of a nursing technical course, how teaching and learning methods were developed during professional formation. It is observed, initially, that the teaching method used is essentially traditional, since expository classes predominate in which the teacher is the holder of knowledge and prepares the study guide to facilitate the learning process. This limits the possibility of students recognizing other ways of thinking and acting on a given theme.

It is also noteworthy that the teacher passes the information related to the class on the blackboard, which seems to impose extreme limits on the creativity and proactivity of the student, as well as distancing itself from what is considered reflective pedagogical praxis.

In this context, it can be inferred that these are classes in the “banking” sense that, according to Paulo Freire’s perspective^(15)^, inhibit the capacity for creation, opposing an educational process in which autonomy is built, considering the dynamics of reality, based on motivating experiences in frank interaction between students and teachers^(15)^.

It is added that the visible social transformations and new technologies, such as cell phones and computers with internet access, which are already available to a large part of the population, reflect on the educational system^(16)^. It is necessary to highlight that such resources can both be used as devices that favor the construction of knowledge and function as a distractor from the teaching and learning process in the face of a mechanical and repetitive teaching model.

Still with characteristics of traditional teaching, it is found in the interviewees’ speeches that the teaching of basic techniques in an isolated and decontextualized way is perpetuated. Even though training in these procedures represents an important aspect in the formation of the nursing technician, at this moment, it is possible that the student cannot articulate the necessary framework of knowledge required for its execution, as the necessary interdisciplinary nature present in this type of activity is not considered. Interdisciplinarity comprises an emphasis on human relationships, based on a pedagogical practice and an appropriate curricular organization, which is in line with the logic of an integrating, active, and participative learning^(17)^.

Regarding the relationship between theory and professional practice, an essential condition for the education of health professionals to work within the logic of the current healthcare model, the interviewees recognize that practice differs from what was seen during the course. To achieve this connection, it is assumed that training institutions and health services work in close partnership. However, despite existing legislation that guides integration between education and service, there is a mismatch between the two, as teachers and students, even when entering health services, remain distant from the realities and obstacles of the profession, maintaining an idealized position that is not considered by service providers^(17-18)^.

Therefore, there is a need for teacher training so that they can adopt a different approach to the learning process, guiding students to understand the dynamics and complexities of care situations as they arise in professional practice^(18)^.

It is common, especially in secondary-level nursing education, to observe that teachers, despite being chosen for their technical excellence in professional practice, lack continuous, interactive, and reflective pedagogical training. Furthermore, as professionals involved in healthcare delivery and management, they incorporate the experiences of the workplace and their own training, leading to the maintenance of traditional teaching principles and the fragmented biomedical care model^(19)^.

To work in vocational nursing education, pedagogical training is necessary so that the teacher can identify how teaching models differ in their basic principles and objectives and direct their teaching practice towards the articulation of theory and practice, with a view to problem-solving in a reflective, creative, and transformative manner^(20)^.

Regarding teachers in technical nursing courses, there is still the realization that they are often inserted into the teaching activity with temporary contracts and part-time workloads, lacking structure for the necessary immersion in teaching activities and for comprehensive and cohesive training, as well as for the articulation of multiple knowledge required to face complex situations^(21)^.

It is necessary to consider that the proposed transformations in the training of healthcare professionals refer to concepts and practices deeply rooted in the social imaginary, which means the existence of a structure of understanding that is shared by the people of a community and, thus, conditions common sense, organizes social relations and practices into legitimate and illegitimate ones, and determines standards considered appropriate or not for that community^(22-23)^.

In light of this, it can be inferred that promoting changes in professional education demands significant institutional investment and openness from the actors involved in the teaching process, as it involves paradigmatic changes in both teaching and healthcare practices. It is therefore a journey marked by a back-and-forth movement, in a dialectical perspective that considers contradiction as the interpenetration of opposing poles, such that the existence of one pole is always conditioned by the existence of the other^(24)^.

The findings of this study are consistent with those of a study conducted with nursing technical teaching staff who also predominantly use traditional teaching methods. In this research, it was found that although they are able to identify the principles of active learning methods and seek to implement teaching strategies that aim to enable greater interactivity, protagonism, and critical thinking in students, indicating that even if slowly, the process of change is being pursued^(25)^.

Part of this movement for change is due to the Curricular Guidelines, which direct the use of integrated curricula, active methods, and the integration of theory with professional practice. Changes in health education are also reinforced by legislation instituted in the sector after the promulgation of the Federal Constitution, which proposed a care model based on the comprehensiveness of care, prioritizing actions focused on primary care in the logic of health promotion and surveillance. Legal provisions therefore constitute an important mobilizer for change, as they encourage actors involved in the process, especially teachers and institutional managers, to engage in reflections on the possibilities of progress^(26)^.

Thus, the need to broaden the debate on curricular guidelines for nursing technician education is emphasized, so that the curricula developed align with them, by strengthening the use of active methodologies, considering their potential for necessary innovation in the teaching and learning process.

Therefore, it is possible to affirm that the implementation of strategies aimed at stimulating student protagonism and proactivity, as mentioned by the interviewees, represents progress in education, as they seek to overcome the fragmented education present in traditional teaching processes, through anchoring in a model that promotes reflection and problematization in relation to healthcare work for understanding the contradictions of professional practice^(27)^.

### Study limitations

This study should be considered in relation to its methodological limitations, as it was conducted in a single institution and, by using a qualitative approach, does not allow for the generalization of results to other populations or contexts.

### Contributions to the nursing field

The results contribute to reflections on a relevant topic for the context of health education and, more specifically, for the education of nursing technicians.

## FINAL CONSIDERATIONS

Our data allow us to conclude that the education of nursing technicians presents significant challenges, as predominantly used teaching methods do not correspond to the needs of the current complex and dynamic reality, especially regarding the production and incorporation of new knowledge into daily practice. In this scenario, graduates value the practical experience of teachers and the introduction of differentiated strategies, such as case studies. Although the introduction of new teaching methods and strategies may be seen as challenging, graduates understand them as interesting and even suggest the use of innovative forms of teaching that enable student proactivity and integration with professional practice. Therefore, it is up to managers to provide structural conditions and support for teacher development, with the aim of incorporating strategies that aim for better alignment of learning methods with the formation of a professional that meets the needs of health services. As for teachers, it is their responsibility to incorporate more suitable pedagogical principles for a technological world, characterized by complexity and dynamism.

## Data Availability

https://doi.org/10.48331/scielodata.DHRP4Y

## References

[1] Camargo RAA, Gonçalves AE, Góes FSN, Nakata CY, Pereira MCA. (2015). Assessment of the training of nursing technicians by nurses who work in hospitals. REME Rev Min Enferm.

[2] Presidência da República (BR) (1986). Lei no 7.498, de 25 de Junho de 1986. Regulamentação do exercício da Enfermagem.

[3] Bassinello G, Bagnato M. (2009). Os primórdios do Projeto Larga Escala: tempo de rememorar. Rev Bras Enferm.

[4] Ministério da Saúde (BR) (2006). Profissionalização dos Trabalhadores da Area de Enfermagem.

[5] Evangelista JP, Medeiros MVJ, Camara MAO, Lopes RVN. (2022). O Setor Privado na Educação Básica Brasileira: espaços e mecanismos de participação. Rev Human Inov.

[6] Ministério da Educação (BR) (2021). Conselho Nacional de Educação. Conselho Pleno. Resolução CNE/CP no 1, de 5 de janeiro de 2021. Define as Diretrizes Curriculares Nacionais Gerais para a Educação Profissional e Tecnológica.

[7] Santos DFA, Castaman AS. (2022). Active methodologies: a brief conceptual presentation and its methods. LNH.

[8] Fabbro MRC, Salim NR, Bussadori JCC, Okido ACC, Dupas G. (2018). Active teaching and learning strategies: perceptions of nursing students. Rev Min Enferm.

[9] Mazur E. (2015). Peer Instruction: a revolução da aprendizagem ativa.

[10] Pertille F, Dondé L, Oliveira MCB. (2020). Formação profissional de nível médio em enfermagem: desafios e estratégias de ensino. J Nurs Health.

[11] Braun V, Clarke V. (2006). Using thematic analysis in psychology. Qual Res Psychol.

[12] Souza VRS, Marziale MHP, Silva GTR, Nascimento PL. (2021). Translation and validation into Brazilian Portuguese and assessment of the COREQ checklist. Acta Paul Enferm.

[13] Minayo MCS. (2017). Sampling and saturation in qualitative research: consensuses and controversies. Rev Pesqui Qual.

[14] Costa AP, Amado J. (2018). Análise de conteúdo suportada por software. Rev Lusofona Educ.

[15] Freire P. (1987). Pedagogia do oprimido.

[16] Freitas DA, Santos EMS, Lima LVS, Miranda LN, Vasconcelos EL, Nagliate PC. (2016). Saberes docentes sobre processo ensino-aprendizagem e sua importância para a formação professional em saúde. Interface (Botucatu).

[17] Perez OC. (2018). O Que é interdisciplinaridade? definições mais comuns em artigos científicos brasileiros. Interseções Rev Estud Interdiscip.

[18] Ministério da Educação (BR) (2015). Ministério da Saúde. Portaria Interministerial no 1.127 de 04 de Agosto de 2015. Diretrizes para a celebração de Ação Pública Ensino-Saúde (COAPES), para o fortalecimento da integração entre ensino, serviços e comunidade no ambito do Sistema Único de Saúde (SUS).

[19] Siqueira MCG, Leopardi MT. (2016). O processo ensino-aprendizagem na formação de trabalhadores do Sus: reflexões a partir da experiência da Etsus. Trab Educ Saúde.

[20] Engers PB, Soares RG, Copetti J, Ilha PV. (2022). O arco de maguerez como proposta metodológica para formação em educação em saúde. Vivências.

[21] Fontana PM, Pinto AAM, Marin MJS. (2021). Pontos e contrapontos no desenvolvimento da interdisciplinaridade na formação técnica em enfermagem. Rev Esc Enferm USP.

[22] Pessoa APN, Nogueira FP, Noronha JC. (2020). A importância do imaginário social para a construção das práticas de ensino-aprendizagem contemporâneas. Rev Científ Multidisc Núcleo Conhecimento.

[23] Ramos RK, Souza MIM. (2021). Teachers’ Education and Social Imaginary. Pro-Posições.

[24] Marquit E. (1996). Contradição na dialética e na lógica formal. Princípios.

[25] Damini NMAV, Pinto AAM, Marin MJS. (2021). Use of active methods in nursing technical education. REME Rev Min Enferm.

[26] Presidência da República (BR) (1988). Constituição da República Federativa do Brasil de 1988.

[27] Góes FSN, Côrrea AK, Camargo RAA, Hara CYN. (2015). Learning needs of Nursing students in technical vocational education. Rev Bras Enferm.

